# Artificial Intelligence in Urolithiasis Imaging and Intervention: A Narrative Review of Current Applications, Barriers, and Future Directions

**DOI:** 10.7759/cureus.97716

**Published:** 2025-11-24

**Authors:** Mohammad Ekhlasur Rahman, Mahabub Hassan, Kashif Waheed, Faisal Haque, Nazeer Ibraheem, Muhammad Rakib Hasan, Mohamed Mohamed

**Affiliations:** 1 Urology, West Hertfordshire Teaching Hospitals NHS Trust, Watford, GBR; 2 Urology, Epsom and St Helier University Hospitals NHS Trust, London, GBR; 3 Urology, Broomfield Hospital, Mid and South Essex NHS Foundation Trust, Chelmsford, GBR; 4 Urology, New Cross Hospital, The Royal Wolverhampton NHS Trust, Wolverhampton, GBR; 5 Urology, Watford General Hospital, West Hertfordshire Teaching Hospitals NHS Trust, Watford, GBR; 6 Radiology, Kettering General Hospital, Kettering, GBR

**Keywords:** ai, artificial intelligence, deep learning, kidney stone, kidney stone disease, ksd, urolithiasis

## Abstract

The treatment of urolithiasis is changing quickly by moving away from conventional diagnostic techniques and toward more complex, data-driven strategies. A major part in this change is being played by artificial intelligence (AI) through providing the clinicians with invaluable assistance. This study examines the state of AI applications in urolithiasis today and how they affect everything from treatment planning to initial imaging. AI models are improving the accuracy of computed tomography (CT) and ultrasonography (US) in diagnoses. These techniques provide automatic stone detection throughout the diagnostic process and a precise stone burden calculation, and even assistance in differentiating difficult mimics, such as ureteral stones, from phleboliths. Additionally, sophisticated algorithms and radiomics are demonstrating great promise in determining the composition of stones preoperatively from imaging data or even digital photos. AI has also changed and improved the intervention for kidney stones, which is highlighted by models now capable of predicting the success of procedures like extracorporeal shock wave lithotripsy (ESWL) and percutaneous nephrolithotomy (PCNL), in some cases outperforming traditional scoring systems. Despite this progress, significant hurdles remain, particularly the need for large datasets and ensuring models are reliable and generalizable across different clinical settings. Successfully integrating these powerful tools into daily urological practice will require a concerted effort toward developing best-practice guidelines, robust training programs, and strong interdisciplinary collaboration. This review aims to summarize current AI applications in imaging and intervention for urolithiasis, identify limitations, and outline future research directions.

## Introduction and background

Urolithiasis is becoming a more serious health issue, in which age, sex, ethnicity, and geographic location are some of the elements that contribute to its rising yearly occurrence, which has a substantial impact on health and well-being [1]. Kidney stone disease (KSD) is often discovered in clinical settings by routine diagnostic procedures like X-rays, ultrasonography, and computed tomography (CT) scans [2]. Most KSD patients often do not exhibit any symptoms and may not require any special care [3,4].

Artificial intelligence (AI) in healthcare refers to data-driven applications, algorithms, and equipment that assist physicians by examining sizable, intricate medical datasets. By identifying patterns and correlations earlier than with conventional methods, it facilitates quicker, more accurate decision-making and treatment selection. AI enhances research and development as well as operational efficiency in addition to patient care. The dramatic increase in AI care delivery, investment, and clinical acceptance is proof that AI improves these areas [5,6].

The healthcare sector is increasingly utilizing AI to handle and enhance data collection and usage. This trend is fueled by the swift expansion of medical digitalization and the dramatic rise in data acquisition [7-10]. Machine learning improves the accuracy of diagnoses and reduces human mistakes in several medical specialties by making the best use of medical imaging methods such as ultrasound elastography (UE), CT scans, and magnetic resonance imaging (MRI) [11]. The benefits of AI in urology are also widely covered and highlighted in the body of current scholarly literature [6,12].

## Review

Improved imaging for urinary stones

Computed Tomography

The literature emphasizes that CT is a crucial diagnostic tool for KSD, accurately determining both the location and size of stones. Additionally, cross-sectional imaging can be conducted with lower exposure to ionizing radiation [13]. CT scans are particularly well-suited for machine learning (ML) because they produce high-resolution images. These images enable accurate identification and measurement of stone characteristics, such as shape, texture, and density. The detailed imaging data reveal information about stones that may not be visible to the naked eye. To assist in managing urolithiasis, several CT-based ML models have been developed to automate the detection of stones and quantify the stone burden. This technology can provide valuable support to urologists during both the diagnostic and interventional processes [13-16].

AI can enhance diagnostic accuracy and efficiency while alleviating radiologists' workload by automating routine interpretation and triage steps. For example, Parakh et al. developed a cascade dual convolutional neural network (CNN) architecture tailored to enhance automated kidney stone identification and urinary tract characterization on non-contrast CT [17]. Their dataset comprised 535 scans that were classified by stone size after applying standard preprocessing to improve comparability across images. The workflow was explicitly staged: the first CNN identified the relevant urinary tract anatomy, and the second CNN then detected the presence of stones within those regions, reducing false positives from surrounding structures. This cascading design delivered consistent, high-quality results, achieving strong discriminative performance (area under the curve (AUC) = 0.954). In practice, such systems can surface likely positives sooner, standardize measurements, and decrease time spent on repetitive tasks, allowing radiologists to focus on complex decision-making [17].

It can be challenging to distinguish between pelvic phleboliths and ureteral stones on CT scans since they both show up as tiny, calcified entities with comparable features. Due to a lack of distinct anatomical cues to distinguish them from nearby soft tissue or blood vessels, distal ureteral stones on non-contrast CT provide an even larger challenge. Jendeberg et al. created and verified a CNN that demonstrated a 94% sensitivity, 90% specificity, and 92% accuracy after being trained on 384 pelvic calcifications and evaluated on 50 stones and 50 phleboliths. This performance outperformed a semi-quantitative approach that used attenuation values and volume (49%) and the accuracy of the typical radiologist (86%) [18].

ML models are also being developed to enhance the characterization of critical imaging parameters for stone surgery. For example, a previous study developed a DL algorithm using a multi-stage CNN and thresholding techniques. This algorithm could automatically detect and classify stones on CT scans according to the STONE nephrolithotomy scoring system. The algorithm demonstrated a high sensitivity of 95.9% for overall stone detection and showed strong agreement with radiologist evaluations for stone size, tract length, the number of affected calyces, and stone density [19]. A CNN was created by Cumpanas et al. to automatically segment kidney stones and calculate their volume. With great pixel overlap (R = 0.98, Dice score = 0.9), their AI model correctly calculated stone volume after being trained on 322 non-contrast CTs and verified against a 3D model [20].

Ultrasound (US) and X-rays

Divya Krishna et al. proposed a computer-aided detection system implemented on field-programmable gate arrays to identify renal abnormalities on ultrasound images [21]. Their workflow involved manual selection of the region of interest, followed by image preprocessing for noise reduction. The segmented kidney region was then analyzed by extracting quantitative features, including intensity histograms and Haralick texture descriptors derived from gray-level co-occurrence matrices (GLCM). Initially, a look-up database was used to distinguish normal from abnormal kidneys. Subsequently, in images classified as abnormal, a trained support vector machine (SVM) combined with a multilayer perceptron (MLP) classifier differentiated renal cysts from stones. This method achieved excellent performance on renal ultrasound, with an accuracy of 98.1%, sensitivity of 100%, and specificity of 96.8% [21].

Balamurugan and Arumugam [22] developed a method to predict kidney disease using an artificial neural network (ANN) and US images. They used 750 kidney US images, with 80% for training and 20% for testing, classifying them into normal, cyst, stones, and tumor categories. Implemented in MATLAB, their technique involved preprocessing images, extracting GLCM features, and selecting key features using an oppositional grasshopper optimization algorithm. The ANN then classified images as normal or abnormal, achieving a maximum accuracy of 95.83% and a maximum specificity of 97.22% [22].

Selvarani and Rajendran [23] proposed a novel approach for detecting renal calculi in US images, which often suffer from speckle noise. Their method utilizes an adaptive mean median filter for noise removal, K-means for segmentation, and a meta-heuristic SVM classifier. Tested on 250 real-time clinical images, the proposed AMM-PSO-SVM model achieved 98.8% accuracy, outperforming conventional methods [23].

A computer-aided detection system based on deep learning (DL) was created by Kobayashi et al. to recognize radio-opaque urinary tract stones on X-rays [24]. This system made use of a 17-layer residual network that was evaluated on 190 photos after being trained on 827. To show the likelihood of stone existence at each pixel, it produced heat maps. The system's positive predictive value (PPV) was 66.2%, its sensitivity was 87.2%, and its F-score was 0.752. Notably, the sensitivity and PPV were lower for mid-ureter stones but greater for proximal ureter stones, reaching 92.5% and 87.6%, respectively. The model was effective. It predicted each X-ray in less than 110 milliseconds [24].

Aksakallı and colleagues [25] developed and evaluated various ML and DL models to identify kidney stones on X-rays. They created a dataset of 221 images, divided into 80% for training and 20% for testing. After converting all images to standardized grayscale, they extracted pixel intensity values as features. To address class imbalance, they used over- and undersampling, which improved model performance. They tested several models, including decision trees, random forests, SVM, MLP, k-nearest neighbor (KNN), naive Bayes, and a CNN-based deep neural network, with the F1 score as the primary metric. The decision tree model achieved the best performance, with an F1 score of 85.3% using synthetic minority oversampling [25].

Identification of stone type

To inform treatment choices, radiomics features are being used to help determine the composition of kidney stones, especially before surgery. Cui et al., for instance, used bagged trees and an ensemble learning technique to create a radiomics signature [26]. Using this characteristic, they classified 157 patients' non-contrast CT scans as either having infection stones (98 patients) or not (59 patients). A total of 27 highly predictive radiomics features were chosen using the LASSO (least absolute shrinkage and selection operator) algorithm. With a receiver operating characteristic (ROC) value of 0.97, this model detected kidney stone infections with 90.7% accuracy, 85.8% sensitivity, and 94.0% specificity [26].

Zheng et al. developed a model incorporating radiomics signatures from CT images of 1198 urolithiasis patients [27]. They selected 24 optimal radiomics features from an initial 1316 using LASSO. This model showed strong performance in both training and validation cohorts, with AUC values ranging from 0.812 to 0.898. Importantly, the model significantly outperformed traditional indicators like urine pH, white blood cell count, urine nitrite, and the presence of urease-producing bacteria in detecting infected renal stones (p < 0.001) [27].

The most prevalent kind of stones in clinical practice, calcium oxalate monohydrate (COM) stones, were the subject of an investigation by Tang et al. [28]. From preoperative non-contrast CT scans of 337 COM and 107 non-COM calculi, they retrieved 1218 radiomics characteristics. For the AI model, eight features with non-zero coefficients were chosen using LASSO. When predicting COM versus non-COM stones before surgery, this model showed an accuracy of 88.5%, sensitivity of 90.5%, and specificity of 84.3%. The training cohort's AUC value was 0.935 (95% CI: 0.907-0.962), whereas the testing set's was 0.933 (95% CI: 0.893-0.973) [28].

Hameed et al. developed a deep learning convolutional neural network (DLCNN) that uses radiomics features to predict calculus type with 87% accuracy. The specificity for different calculus types ranged from 85% to 93%. While there have been advancements in using AI, such as distinguishing between pelvic phleboliths and ureteric calculi and incorporating anatomical location, AI used in isolation still has significant inaccuracies. Both AI and radiomics are well-suited for processing large datasets, and the growing availability of big data from electronic patient records will allow them to identify new diagnostic and treatment patterns in the future [29,30].

**Figure 1:**
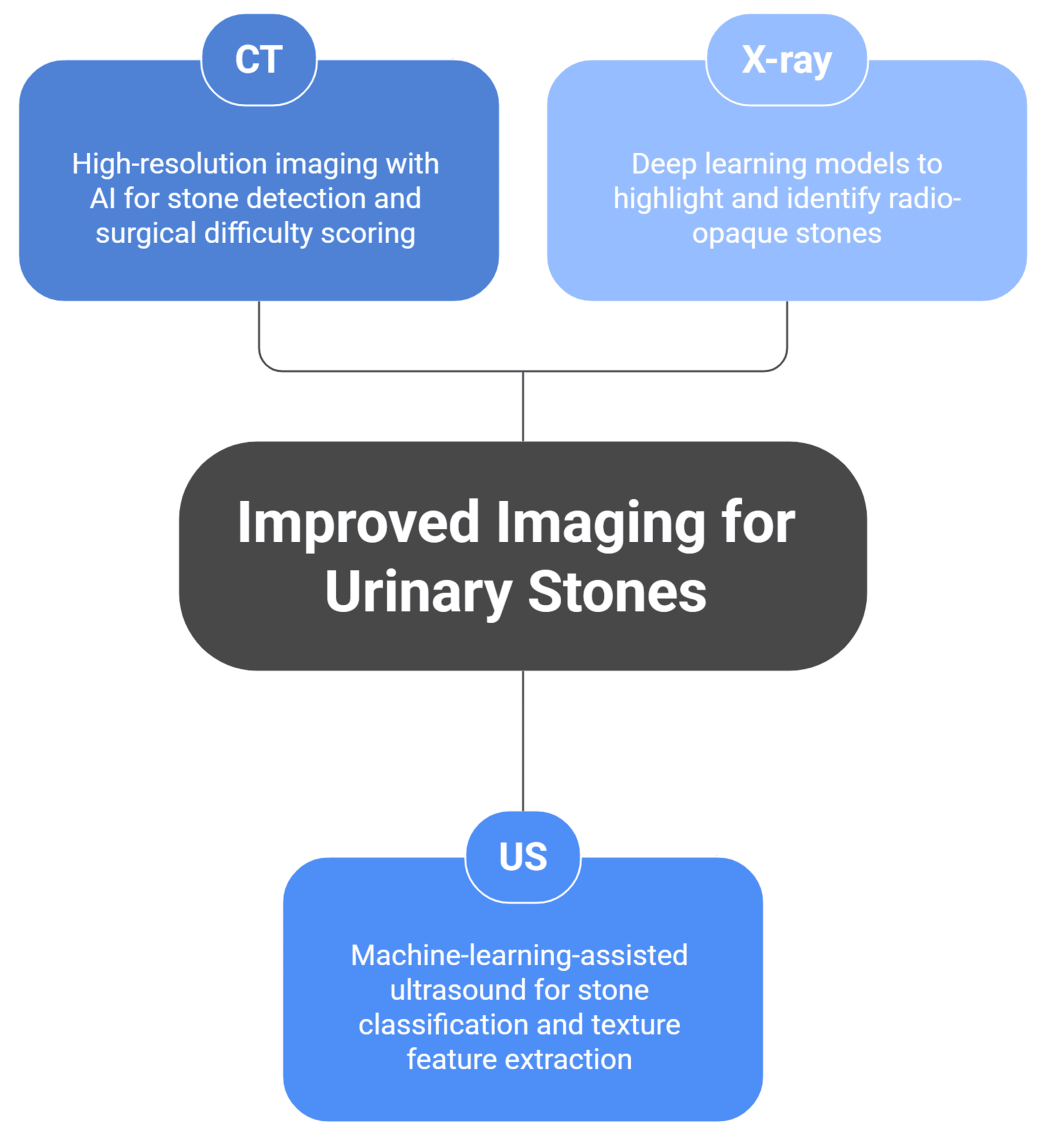
The role of AI in improving imaging for urinary stones. This figure was designed by the article authors based on references [13-25]. CT: computed tomography; US: ultrasound.

The ability of a DL technique to identify kidney stone composition from digital photos was evaluated by Black et al. [31]. Five distinct compositions of human kidney stones, including COM, uric acid, struvite, brushite, and cystine, were used in the investigation. Surface and inner core images were evaluated by a deep CNN (ResNet-101). The model obtained an overall weighted recall of 85% for composition prediction using leave-one-out cross-validation. The highest recall rates were for calcium oxalate (90%) and uric acid (94%) stones. The authors came to the conclusion that DL can accurately recall the composition of stones, pointing to possible future uses for autonomously guiding laser settings during endoscopy (Figure 1) [31].

AI in the treatment of kidney stones

Significant technological breakthroughs have been made in KSD management. Using information from 384 patients with stone formation, including those who passed stones naturally or who received treatments (stent, ureteroscopy, or extracorporeal shock wave lithotripsy (ESWL)), Parekattil et al. created a model. According to their research, patients who might need intervention can be reliably identified using a stone dimension threshold of 6 mm [32].

A substantial body of research has employed ANNs to forecast treatment outcomes in KSD, with the overarching aim of preventing or at least reducing burdensome stone recurrence. These models are typically trained on rich, multimodal inputs that reflect the patient’s clinical profile, such as demographics, comorbidities, prior stone history, imaging findings, and peri-procedural details, alongside biochemical information obtained from urine samples. In the specific context of predicting post-ESWL recurrence, studies frequently assemble cohorts of patients who have undergone ESWL and use their pre- and post-procedure clinical data and urinary analytes as predictive factors. The ANN then learns patterns that correlate with the likelihood of recurrence, enabling risk stratification, informing follow-up intensity, and supporting individualized counseling without altering the underlying clinical decision pathways [33,34].

Poulakis et al. demonstrated that ANNs can accurately predict stone clearance after ESWL [35]. Their retrospective study of 680 patients found that radiographic images, categorized by their morphological patterns, could predict successful stone clearance (defined as no fragments at six months) with 92% accuracy. The most influential predictors were the pattern of dynamic urinary transport, followed by the infundibuloureteropelvic angle, body mass index, caliceal pelvic height, and stone size. While highlighting the high accuracy of ANNs in predicting shock wave lithotripsy (SWL) outcomes, the authors emphasized the need for prospective validation of these findings [35].

Mannil et al. evaluated whether ML and 3D texture analysis (TA) of CT scans might forecast the success of SWL [36]. They discovered that typical clinical indicators and stone Hounsfield unit (HU) values showed limited predictive power (AUC = 0.58-0.68) after analyzing 224 TA characteristics and clinical data (BMI, stone size, and skin-to-stone distance) from kidney stone patients. An AUC of 0.79 was attained by a random forest model that only used three 3D TA characteristics. The discriminatory accuracy (AUC = 0.85) was further enhanced by combining these TA features with clinical data, such as skin-to-stone distance, suggesting that 3D TA offers additional prognostic information [36].

Moorthy et al. used non-contrast computed tomography (NCCT) image analysis to predict renal stone fragmentation after ESWL [37]. To predict breakability, the researchers employed a neural network to extract first-order statistical (FOS) properties from the NCCT pictures of 120 patients. The prediction model achieved an overall accuracy of 90% by predicting fragmentable stones (true positives) with a sensitivity of 80.7% and non-fragmentable stones (true negatives) with a specificity of 98.4% using the "mean" FOS feature alone. The scientists came to the conclusion that the results of ESWL treatment may be reliably predicted using this statistical and neural network technique [37].

Choo et al. developed a decision support system (DSS) that, particularly with their 15-factor model, combines stone features from CT and X-ray images to reliably predict the outcome of ESWL treatment [38]. Yang et al. also discovered that a DSS could accurately predict ESWL success rates up to 88% of the time [39]. More recently, an ML model that helped practitioners make decisions was able to predict ESWL outcomes with a sensitivity of 87.5% [40].

Percutaneous Nephrolithotomy (PCNL)

Aminsharifi et al. were the first to use AI to predict outcomes for percutaneous nephrolithotomy (PCNL). They used an ANN with data from 200 patients to identify factors predicting overall outcomes like stone-free rate (SFR) and the need for blood transfusions. The AI system, tested on 254 patients, showed high sensitivity in predicting PCNL outcomes: 92% for SFR, 97% for repeat PCNL, 82% for additional shockwave lithotripsy, and 91% for additional ureteroscopy. With an overall AUC of 0.915, the AI outperformed existing scoring systems like Guy’s Stone Score and the Clinical Research Office of the Endourological Society (CROES) nomogram in predicting post-PCNL SFR [41,42].

**Figure 2:**
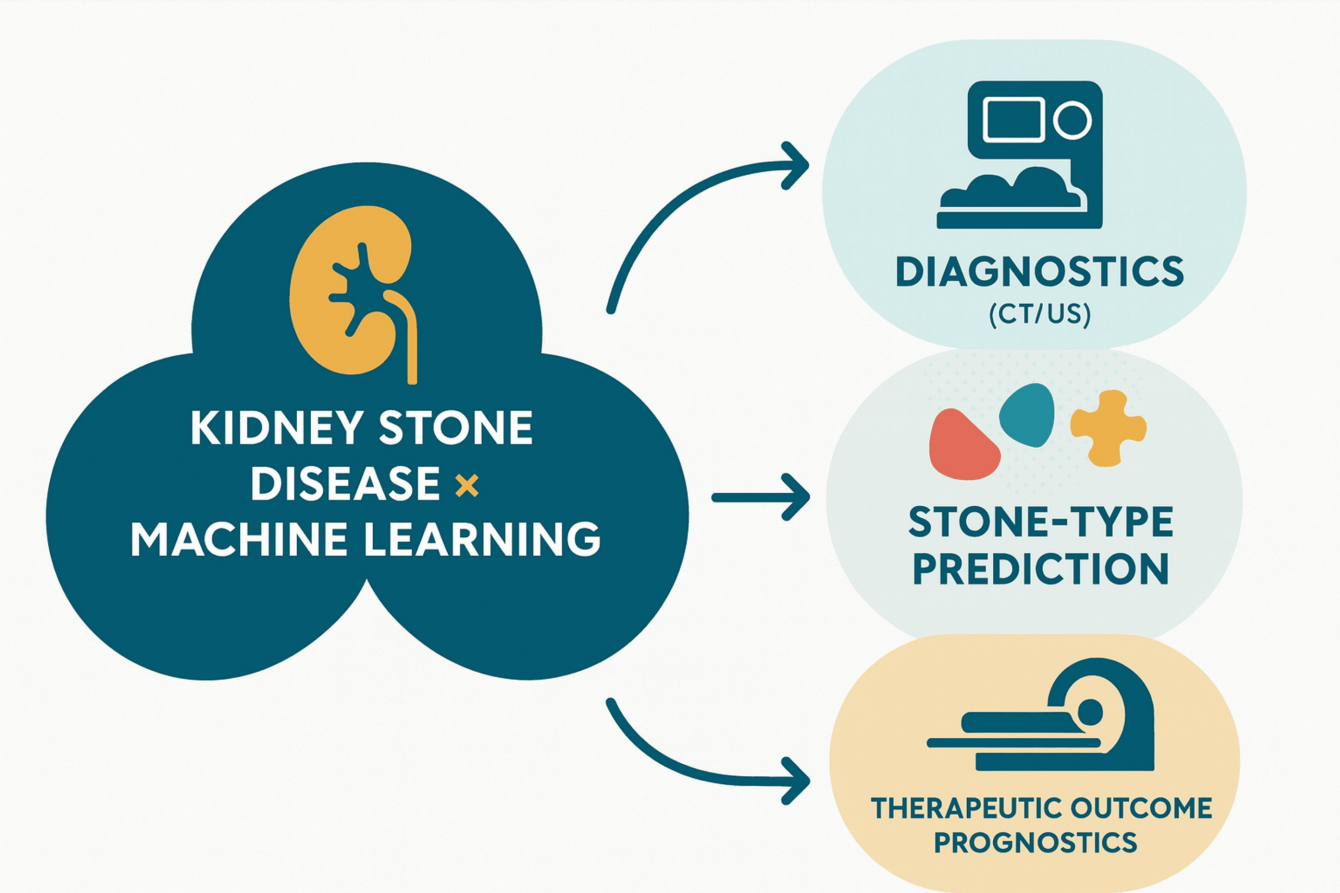
The role of AI in kidney stone disease (KSD). This figure was created by the article authors summarizing the benefits of AI in KSD [13,26,31,35]. CT: computed tomography; US: ultrasound.

A DSS was created by Shabaniyan et al. to help with PCNL treatment of big kidney stones [43]. They entered CT features and other patient and perioperative data from 254 instances into an ML system. All patients had postoperative CT scans to determine their SFR. The DSS predicted postoperative outcomes with an overall accuracy of 94.8%. In particular, it had a sensitivity of 80% to 90.9% for predicting the need for additional procedures (PCNL, ureteroscopy, SWL). Additionally, the DSS showed a sensitivity of 85.2% for predicting the need for a blood transfusion and a sensitivity of 71.1% for predicting the need for a stent (Figure 2) [43].

Limitations and future directions

The fact that features are extracted straight from the input data, establishing a dependent relationship, is a disadvantage of DL radiomics. This implies that huge datasets are essential for precisely finding strong and pertinent feature subsets, in contrast to feature-based radiomics. One ML technique that can help with this problem is transfer learning. This method makes use of a neural network that has already been trained on a related task; for instance, a neural network that has been trained to identify kidney stones can be further modified to measure and classify any leftover pieces following surgery [44].

Additionally, a major challenge is the generalizability and dependability of radiomics properties. Many aspects of image capture and processing have a significant impact on their integrity. These include the image's size and general quality, the particular scanning sequence used, the imaging modality, the resolution attained, and whether motion artifacts were present during image transfer. These relationships were carefully examined in a recent review by Traverso et al., which identified particular radiomics properties that continuously showed consistency and repeatability under a variety of settings [45].

Future Directions

Urology is a medical field that has always advanced quickly by incorporating new technologies. This embrace of cutting-edge tools and methods is intended to enhance patient care and produce better results [46].

In contrast to previous methods, AI techniques have the potential to drastically change how urolithiasis cases are managed [47]. From diagnosis and treatment planning to follow-up and prevention, these cutting-edge methods have the potential to completely transform patient care, ultimately resulting in more individualized and successful treatments.

An ensemble learning-based DSS for early detection and categorization of kidney stones was developed by Kazemi et al. [47]. Using a variety of AI algorithms, including Bayesian models, decision trees, ANNs, and rule-based classifiers, this system achieved 97.1% accuracy. These algorithms were utilized to decipher complex biological characteristics associated with kidney stone development prediction and type determination [47].

Längkvist et al. created a CNN model that successfully identified ureteral stones in high-resolution CT scans, achieving perfect specificity and an AUC-ROC of 0.9971, with a false positive rate of 2.68 per scan [15]. The successful integration of AI into clinical urology will require urological societies to establish best practice guidelines, develop AI training for healthcare providers, create new urology-specific AI tools, and encourage better collaboration between academic institutions, hospitals, AI technology firms, government bodies, and local communities.

## Conclusions

AI is rapidly becoming an integral partner in the diagnosis and management of urolithiasis. Across CT, ultrasound, and X-ray imaging, AI models can automatically detect stones, quantify stone burden, distinguish ureteric calculi from common mimics, and even infer stone composition using radiomics or digital photographs. In the interventional domain, ML and neural network-based decision support systems already match or outperform traditional scoring tools for predicting outcomes after ESWL and PCNL, supporting individualized treatment selection and more efficient use of resources. Together, these advances suggest that AI can enhance precision, streamline workflows, and potentially improve patient counseling and follow-up in stone disease.
